# Vascular labeling of the head and neck vessels: Technique, advantages and limitations

**DOI:** 10.4317/jced.53832

**Published:** 2017-05-01

**Authors:** Alba Gálvez, José-Leonardo Caraballo, María-Cristina Manzanares-Céspedes, Iván Valdivia-Gandur, Rui Figueiredo, Eduard Valmaseda-Castellón

**Affiliations:** 1 Oral Surgery and Implantology Master degree program; Faculty of Medicine and Health Sciences, University of Barcelona, Spain; 2 Human Anatomy and Embryology Unit. Experimental Pathology and Therapeutics Dpt, University of Barcelona (Spain). Facultat de Medicina. C/ Feixa Llarga, s/n; Pavelló Govern, 5ª planta, 08907 L’Hospitalet de Llobregat; Barcelona, Spain; 3 Human Anatomy Unit, Biomedical department and Odontology department, University of Antofagasta, Chile

## Abstract

**Background:**

Vascular staining techniques have been used to describe the vascular structures of several anatomic areas. However, few reports have described this procedure in the head and neck region. This paper describes a head and neck vascular labeling procedure, and describes some of the technical complications that may occur.

**Material and Methods:**

Fifteen specimen cadaver heads were prepared. After drying the vascular system, the internal carotid arteries were ligated and a solution with latex and a gelling agent was injected into the internal carotid arteries and external jugular veins. Two different colors were employed to differentiate arteries from veins. A total of 60ml latex was injected into each blood vessel. Subsequently, the specimens were refrigerated at 5°C for a minimum of 24 hours. Finally, a dissection was performed to identify the venous and arterial systems of the maxillofacial region.

**Results:**

In most specimens, correct identification of the vascular structures (lingual artery, pterigoyd plexus, and the major palatal arteries, among others) was possible. However, in three heads a major technical problem occurred (the latex remained liquid), making the dissection unfeasible. Other minor complications such as latex obstruction due to the presence of atheromas were found in two further specimens.

**Conclusions:**

The vascular labeling technique is a predictable, effective and simple method for analyzing the vascular system of the maxillofacial area in cadaveric studies, including vessels of reduced diameter or with an intraosseous course. This procedure can be especially useful to teach vascular anatomy to dental students and postgraduate residents.

** Key words:**Blood vessels, vascular casting, vascular labeling, head and neck arteries, carotid arteries, jugular veins.

## Introduction

Vascular staining or labeling techniques have been used since the sixteenth century to describe vascular structures (1,2). These techniques are based on fixing and filling the vascular structures through step-by-step preparation of the cadaveric material. In all dental specialties, knowledge of the vascular anatomy of the region is paramount to avoid intraoperative complications. Therefore, labeling techniques can be quite useful for training undergraduate and postgraduate students.

The vessels of the limbs have been widely studied using these and other well-known procedures (2). The Spalteholz technique is similar to vascular staining but includes an additional corrosion process to enhance bone transparency in order to view the intraosseous vascular structures. Nevertheless, this method uses peroxide (a classic clearing procedure first introduced by Spalteholz in 1911) (3), which can damage the tissues and therefore hamper any histological analysis. Steinke and Wolff proposed a modified Spalteholz method to maintain the transparency of the specimen without compromising the histological investigation (4). Other techniques used to explore intraosseous blood vessels are more complex and include diaphanization, radiological contrast injection and consecutive radiological examination. This produces detailed results but is time-consuming and can lead to high failure rates (5).

Few studies have applied vascular labeling techniques in the head and neck area (6-8). These methods are able to identify both intraosseous and extraosseous vessels in fine detail. Furthermore, they provide a reliable, realistic and three-dimensional view and make it possible to detect variations that otherwise might remain unnoticed (9). Vascular labeling allows simplifying the classic dissection methods for anatomical study, which can be quite demanding for dental students, residents and clinicians. From a clinical point of view, these kinds of anatomic studies allow dentists and surgeons to plan operations more accurately, since they can practice the procedure in specimens, easily identify vascular structures (even intraosseous vessels) and differentiate arteries from veins (different colored latex solutions can be used) (8).

This paper aims to describe a procedure for head and neck vascular labeling with liquid latex. Furthermore, it will examine the main advantages, complications and limitations of this technique.

## Material and Methods

Fifteen fresh frozen cadavers donated to the Faculty of Medicine Dissection Room and Donation Service of the University of Barcelona were used. The specimens were preserved in the dissection room at -16 °C. After defrosting the cadavers, the large vessels of the head were identified, and their blood was removed.

-Sample preparation: washing and drying of cadaveric specimens

Firstly, using a pump device, the arterial and venous circuits of the head were washed with a mixture of water and embalming solution (phenol 90%: 12.5ml; ethanol 96%: 62.5ml; 35-40% formaldehyde solution: 7.5ml and glycerol 17.5ml), injected through the common carotid artery (CCA) and the internal jugular veins. A total of 12 L of solution were injected into each specimen. Afterwards, ammonia water (0.1% ammonia) was also introduced into the vessels, according to the manufacturer’s instructions. While the vessels were being cleaned, the water pump was stopped every 10-15 minutes and the head was positioned in an upright position. This procedure was repeated at least 3 to 4 times. Subsequently, the heads were dried manually by blowing air into the same blood vessels, and the specimens were placed in an upright position to remove any remnants of the injected solution. This procedure was also repeated several times.

**Figure 1:**
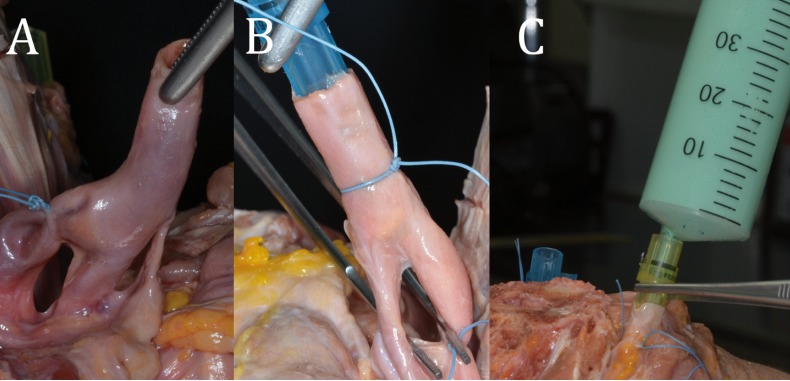
A. Ligation of the internal carotid artery, after dissection of the carotid bifurcation. B. Plastic cannulas placed in the external carotid arteries and internal jugular veins. C. Injection of latex.

-Dissection of carotid bifurcation and ligation of internal carotid:

A careful dissection of the carotid bifurcation was then performed, and the internal carotid artery was ligated with 2/0 silk sutures (Ethicon, Johnson & Johnson, New Jersey, USA) (Fig. 1A). This manoeuvre was performed to avoid the latex going in the wrong direction, and so that the latex would run through the branches of interest. Plastic cannulas were placed in the external carotid arteries and internal jugular veins (Fig. 1B).

-Preparation and injection of latex:

Each vascular structure was injected bilaterally with RV-30L latex (ResinpolLatex Compound SL, Terrassa, Spain), mixed with a gelling agent (GELIF/F). Two different colors were used to differentiate the arterial (blue) and venous (green) structures. A total of 60 ml of latex were injected into each blood vessel (240 ml in total). For every 60 ml of latex, 0.25 ml of the gelling agent (Re-sinpolLatex Compound SL, Terrassa, Spain) were added to prepare the latex mixture. Hypodermic syringes were used to inject it into each blood vessel (Fig. 1C). The fresh specimens were preserved at a constant temperature of +5 °C for a minimum of 24 hours and a maximum of 3 days, especially because the cadavers’ soft tissues are extremely sensitive and could decompose very fast, even at low temperatures.

**Figure 2:**
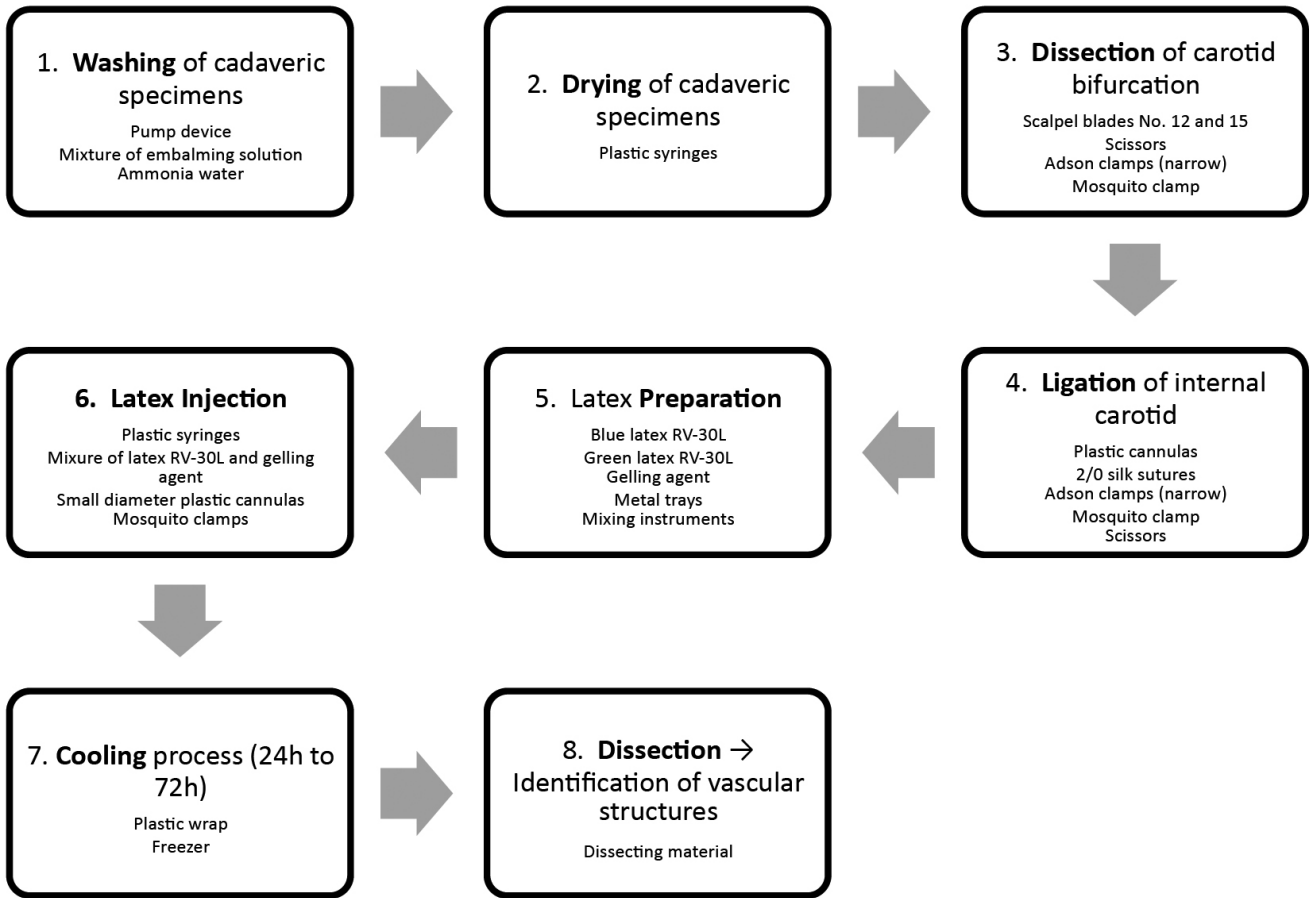
Sample preparation. Main steps in preparing cadaveric material for head and neck vascular labeling with liquid latex, and materials required.

A flow-chart describing the main steps in this technique can be found in figure 2.

-Identification of vascular structures:

Finally, the venous and arterial routes of the oral and maxillofacial region were dissected. Photographic records were made with a reflex digital camera (Nikon SLR D5100, Tokyo, Japan) in the dissection room, in order to illustrate the position and size of the vessels.

Two researchers (LC and AG) recorded the vascular structures identified and the technical complications encountered.

-Statistical analysis

Descriptive analysis was performed using the IBM SPSS Statistics 21.0 software package (IBM Corp.; Armonk, NY; USA).

## Results

**Figure 3:**
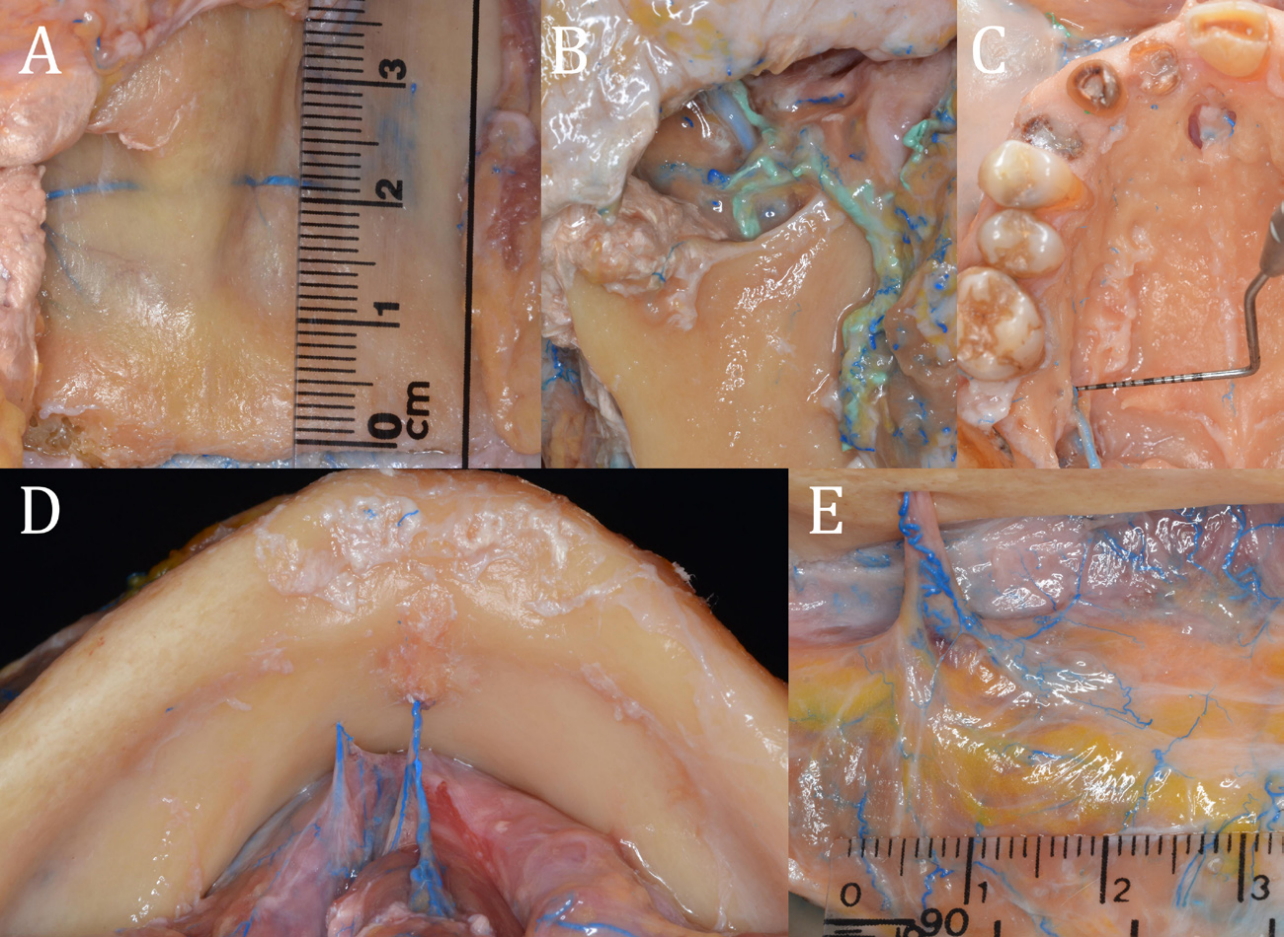
A. Measurements of the posterior superior alveolar artery in the anterior sinus wall. B. Pterygoid venous plexus identified by the vascular labeling technique. C. Major palatal artery exiting the palatine foramen. D. The lingual artery. E. Blood vessels of less than one millimeter in diameter colored by the latex used in the vascular labeling technique (mental foramen).

In total, 15 heads with a mean age of 82 years (range 66-102) were prepared ( Table 1). Correct identification and dissection of the arteries and veins was possible in most of the specimens (Fig. 3). Among the most interesting structures that could be easily identified through this procedure were the lingual artery (Fig. 3D), the pterigoyd plexus (Fig. 3B), the intraosseous branches of the posterior superior alveolar artery in the lateral wall of the maxillary sinus (Fig. 3A), and the major palatal artery (MPA) (Fig. 3C). Small-caliber vessels were also stained, as can be observed in figure 3E (Fig. 3E).

**Table 1:**
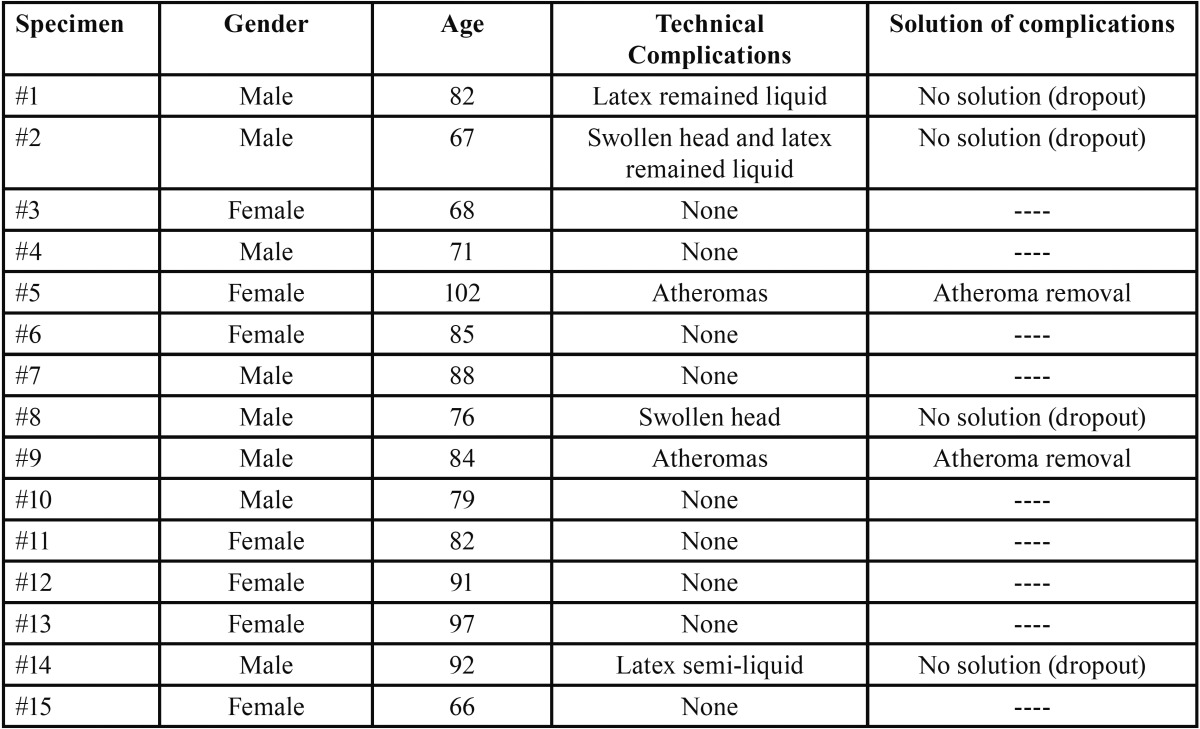
Distribution by gender and age, with technical complications and their solution.

Nevertheless, several technical problems were registered during the present study. In three heads (#1, #2, #14) the latex remained liquid 48 hours after injection, and in another two specimens, atheromas blocked the entrance of the latex into the external/common carotid artery (#5 and #9). While the atheromas were easily removed and did not affect the final result of the dissection, in the cases where the latex remained liquid it proved extremely difficult to perform the dissection. In order to solve the latter complication, specimens #1, #2, #14 were returned to low temperature conditions in an attempt to increase the viscosity of the latex. However, this did not solve or improve the problem.

Another complication was that two heads (#2 and #8) swelled during the process, probably due to a chemical reaction that produced gas inside the vascular structures. This event jeopardized the entire dissection, since many structures could not be identified in these specimens.

## Discussion

Blood vessels are especially important structures, and their identification is mandatory in oral surgical procedures. The vascular staining or labeling technique allows easy identification of vascular structures, even when they measure less than one millimeter in diameter (Fig. 3E). Moreover, this technique detects any anatomic variation in a very reliable way compared to other methods (7,10,11). For these reasons, we believe that vascular labeling is an extremely useful procedure for teaching dental students and postgraduate residents. It might also be interesting in hands-on cadaver courses, since in this way surgical trainees can easily observe and understand the vascular anatomy during simulated surgical procedures.

Latex, epoxy resin or radio contrasts have been proposed as materials for injecting into specimens to display intraosseous blood vessels (12,13). Tetroxide and oxide are examples of the most commonly used radio-opaque injection solutions. Other agents, such as barium sulphate and colloidal silver or mercury, have also been used in femoral and humeral blood vessels (14,15). Latex was chosen for our study because it is easy to handle, affordable, has an acceptable working time, adequate thickness and allows several colors to be employed.

Some critical issues should be taken into account in order to prevent technical complications during specimen preparation with vascular labeling procedures. The correct choice of latex solution is crucial. It must combine the following features: the ability to solidify inside organic materials; an adequate working time (enough to prepare and inject it) and setting time (ideally 24 hours or less, to avoid specimen decomposition), and availability in different colors. Thorough drying of the vessels is also extremely important, since liquids inside the vascular structures can hinder the correct diffusion of the latex. Also, incorrect drying can lead to chemical reactions like those observed in two specimens (#2 and #8) in this study.

A possible limitation of this technique is related to age-dependent anatomical structures. Indeed, most specimens belong to elderly patients, which might limit the generalization of the results. For example, if the location of the vessels is described using certain bone structures (for example, the alveolar bone of the maxilla) as the reference point, the measurements are likely to be useless in younger patients (who have less resorption of the alveolar bone). Also, the vessel diameter and number might be affected by age due to microvascular defects or stenotic changes (16,17).

Some vascular structures can be identified through radiological examination. A retrospective study with computer tomography (CT) images of 200 patients found 236 mandibular lingual canals (MLCs) and 159 lateral lingual canals (LLCs) (18). During dental implant placement, an injury to these vessels can lead to life-threatening complications, such upper airway obstruction, caused by profuse bleeding (19-22). Also, the posterior superior alveolar (PSAA) or infraorbital arteries that might be injured during maxillary sinus augmentation procedures can be seen in CT scans, since these vessels are mostly intraosseous (23). Thus, surgeons can sometimes assess the path of the above-mentioned vessels preoperatively through these images. However, many vascular structures cannot be identified easily in radiological images. In these cases, clinicians might need to use cadaveric material to increase their anatomical knowledge of the area of interest. Classic dissection is the most common tool, but it is demanding and requires experience, knowledge and ability. With dental students and less experienced professionals, some important vascular structures may remain unnoticed. In contrast, labeling techniques allow most arteries and veins to be detected, as observed in our sample.

In short, the vascular labelling technique is a predictable, effective and simple method for analyzing the vascular system of the maxillofacial area in cadaveric studies. If the preparation protocol described is followed exactly, few minor technical complications will occur. Furthermore, this technique makes it easy to identify almost all the vessels of the oral and maxillofacial area, even those of reduced diameter or with an intrabony course.
